# Multi-species entanglements and stable isotope signals (*δ*^13^C and *δ*^15^N) in modern reindeer herding communities of boreal northeast Asia

**DOI:** 10.1098/rstb.2024.0203

**Published:** 2025-05-15

**Authors:** Morgan Windle, Sarah Pleuger-Dreibrodt, Julia K. Clark, J. Bayarsaikhan, William Taylor, Henny Piezonka

**Affiliations:** ^1^ Cluster of Excellence ROOTS, Kiel University, Kiel, Schleswig-Holstein, Germany; ^2^ School of History Classics and Archaeology, University of Edinburgh, Edinburgh, UK; ^3^ Nomad Science, Ulaanbaatar, Mongolia; ^4^ Institute of Archaeology, Mongolian Academy of Sciences, Ulaanbaatar, Mongolia; ^5^ Department of Anthropology, University of Colorado Boulder, Boulder, UK; ^6^ Free University of Berlin Institute of Prehistoric Archaeology, Berlin, Germany

**Keywords:** dietary isotopes, multi-species, North Asia, reindeer domestication, Indigenous Knowledge

## Abstract

Prevailing anthropocentric frameworks of animal husbandry in archaeological research are increasingly critiqued for their inability to capture the full spectrum of human–non-human systems. In west Siberia and northern Mongolia, reindeer herding communities practise an entwined multi-species lifeways with the subarctic boreal and forest ecosystems—but these practices lack secure archaeological chronologies and time depth in northeast Asia. Traces of reindeer herding and reindeer remains themselves are often under-represented in the depositional record, requiring alternative avenues for tracing the archaeology of reindeer herding. Here, we explore the potential of documenting these complex dynamics archaeologically through a proof-of-concept analysis of stable isotopic carbon and nitrogen in faunal bone collagen, which can represent a possible nexus of multi-species practices. In doing so, we seek to expand investigative potentials into both human and non-human community members, providing valuable, nuanced insights into past practices, hunter–herder interactions and domestication dynamics.

This article is part of the theme issue ‘Unravelling domestication: multi-disciplinary perspectives on human and non-human relationships in the past, present and future’.

## 1. Introduction

For the past two centuries, historians, ethnographers and archaeologists have studied how fisher–hunter–gatherer communities in northern Eurasia integrated domestic reindeer (*Rangifer tarandus*) into their lifeways. Extensive work to understand the complex dynamics between humans and reindeer [1–7] and to pinpoint these relationships in the past [8–10] has led to a range of narratives on the origins of reindeer domestication in specific reindeer herding cultures. However, the exact time and place of reindeer domestication remain uncertain [11,12]. Today, reindeer are central to Indigenous fishing–hunting–gathering lifeway in northern Eurasia, as well as parts of Alaska, Canada and Greenland, serving as both domestic animals and wild game and shaping circumpolar societies since the Last Glacial Maximum [13,14].

Scholars increasingly show that domestication is a complex spectrum, with animals acting as agents within this continuum [15–17]. Domestication is a continuing evolutionary process shaped by both natural and human-induced selection, where species can become genetically distinct, more dependent on humans for resources and exhibit phenotypic or behavioural changes [18]. However, it involves not just a genetic transformation but also a coevolutionary relationship, where humans and animals mutually influence each other through ongoing interaction, skills and knowledge [9]. While criteria such as genetic divergence and physical traits are debated as markers of domestication, the process is understood as both a biological and a cultural adaptation [15,16,18,19].

Reindeer are often referred to as ‘semi-domesticated’ [20–22], but this designation implies that domestication is incomplete or reaches a final stage, which does not align with current understandings of domestication (e.g. [15,16]). In reality, domestication is not a fixed endpoint but rather a fluid, ongoing process where humans and animals continue to influence each other’s abilities and survival strategies over time [9,16]. Labelling reindeer as ‘semi-domesticated’ therefore fails to capture this continuous interaction and coevolution. For this reason, here, we will refer to reindeer cohabiting with and being herded by communities as domesticated.

The current perspective on domestication is exemplified in the practices of reindeer herding, where humans and domestic reindeer adjust their respective behaviours to coexist within a shared, multi-species landscape. Reindeer herding often lacks conventional controls, emphasizing mutualism and symbiosis [1,23,24]. Reproductive controls do not follow a standard trajectory, as wild and herded reindeer can be intentionally interbred [25]. Herd composition can be highly variable, as it is not uncommon for working reindeer to join wild herds or vice versa [26]. Herders view domestic reindeer as community members, and ecological knowledge of reindeer dietary habits and behaviours plays a significant role in this mutualistic relationship [2,27], suggesting socio-cultural factors as key drivers in mutualistic systems. As more proxies for understanding past human–animal relationships emerge, such as soil biomarkers [28,29] and pathology analysis [30], it is crucial to integrate the diverse forms of human–reindeer cohabitation into other techniques for understanding the archaeology of domestication. The practices of reindeer herding communities could provide insights into the traceability of past human–reindeer cohabitation, deepening our understanding of animal integration into human lifeways.

Here, we examine the reindeer herding practices of the Sel’kup, and to a lesser extent the Khanty, in west Siberia, Russia, and the Tsaatan of northwest Mongolia. We aim to integrate a multi-species interpretive framework with stable isotope and ethnographic data. In doing so, we expand current biochemical indicators to allow for comparative analyses of archaeological and modern reindeer specimens while also testing the visibility of these mutualistic, multi-species relationships and considering broader implications regarding domestication.

### (a) Multi-species frameworks in archaeology

In archaeological research non-human animals have often been considered through western [31], modern capitalistic [32] and highly anthropocentric lenses [33]. As a result, by studying animals as victims, symbolic objects or products in the archaeological record, they serve more as proxies for human action than representatives of an interspecies relationship [34,35]. The nature of these conceptual frameworks has had tangible impacts on narratives of human/non-human–animal relationships through time, for instance, in contributing to the secondary products’ revolution hypothesis [36,37], dichotomous wild versus domestic categorizations [38,39] and strict economic frameworks, particularly among hunter–fisher–herders [40,41]. Some scholars increasingly critique this framing of non-human animals, as it ineffectually captures the spectrum of roles which non-human animals have played in human societies [42–45].

Human and non-human–animal relationships are rarely two-way exchanges but exist in a network of relationships [46,47]. Newer frameworks examine interspecies rhythms [43,48] and prioritize non-human animals in their conceptual approaches [38,49]. These multi-species perspectives shift focus away from human dominance, situating the human past within a broader ecology of non-human actors and revealing key archaeological insights [43]. For example, isotopic analysis and ancient genomics of woolly dogs, such as those from the Coast Salish in the Pacific northwest, revealed how dogs shared human mobility and diets and participated in cultural practices and shaping human landscapes [50]. Similarly, reconstructing red deer antler use in Mesolithic tools and objects with such an interpretive lens highlighted human–animal entanglements beyond subsistence that contribute to the archaeological record [51]. Recognizing non-human animals as co-creators of the world has significant implications for understanding past societies, but archaeological methods must fully adapt to capture these dynamics.

### (b) Dietary isotopes in human–animal archaeologies

One such analytical method is the use of stable isotopes, a valuable method for reconstructing past populations, migrations and cultural practices, which offers an alternative criterion for identifying domestic reindeer in the archaeological record without requiring robust archaeofaunal assemblages. While various isotopes are used in human–animal relationship reconstructions, such as strontium [52] and oxygen [53,54], stable isotopes of carbon and nitrogen could represent an effective avenue for identifying multi-species practices integral to systems like reindeer herding. Carbon and nitrogen stable isotopes are influenced by environmental factors, allowing for the reconstruction of bulk components in the diet of consumers. Where carbon stable isotopes help distinguish diets based on plant photosynthetic pathways and, to some extent, terrestrial versus marine food sources [55–58], nitrogen isotopes predominantly reveal protein sources in diets but also values influenced by local environmental conditions [59,60].

Generally, *δ*^13^C values of C3 plants are sensitive to a wide range of environmental factors, which for example, can increase *δ*^13^C values by 2−3‰ owing to water stress, high salinity soils and high elevation, whereas C4 plants exhibit slight variation in *δ*^13^C owing to environmental factors [61]. Variation between different organic tissues, different individuals/species and the influence of different diets can lead to larger offsets [62]. In research involving complex environmental food webs, it is the combined analysis with stable isotopic nitrogen which is key [63,64]. Stable isotopic nitrogen compositions of skeletal tissues are particularly impacted by the consumer’s trophic level position, additionally indicating bulk terrestrial versus aquatic dietary components and different environmental factors impacting plants [65,66]. A stepwise enrichment of ^15^N occurs with each upward shift in trophic level in the food chain [57,67], and the influential variables are well documented. Herbivore tissues are typically enriched in ^15^N compared to the plants they consume, with ^15^N levels increasing up the food chain [68]. Aquatic food chains also tend to be longer than terrestrial ones, incorporating a greater number of piscivorous trophic levels and allowing for the detection of aquatic resources as a dietary component in terrestrial consumers [57,69]. The presence of fishes in freshwater environments, such as those in boreal/taiga regions where reindeer are herded and which are vital resources for reindeer herding communities [70], as well as their diet and migration patterns between freshwater bodies, is reflected in the nitrogen values found in fish bone collagen.

The challenge of disentangling carbon and nitrogen isotopic signatures is because different tissues integrate isotopes over varied timescales. This makes tissue choice essential for reconstructing diet and ecology. Bone collagen reflects 10−20 years in an adult mammal owing to slow turnover, while in younger animals, it captures shorter, life-stage-specific periods, such as elevated values from milk intake during preweaning [71–74]. Other tissues like dental collagen, formed early, preserve juvenile dietary information, while antlers and keratin in hair and nails reflect shorter-term dietary inputs, from weeks to months, useful for tracking seasonal or episodic changes [72,74,75]. The animal’s age at sampling is also important, as older animals show broader dietary histories, while younger animals’ values reflect more immediate dietary influences, sensitive to rapid shifts. Both carbon and nitrogen values expressed in different species of wild or domestic herbivores depend on their dietary preferences and the seasonality of available food sources [72,76,77]. Bone collagen serves as an effective starting point for disentangling herded reindeer diets in North Asia, providing a broad baseline for dietary inputs that can reveal overarching trends and ecological and anthropological influences on reindeer populations.

Stable isotope analyses provide versatile tools and concepts that archaeologists, regardless of their theoretical approach, can effectively use [78]. A pluralistic approach in archaeology integrates diverse frameworks and bridges research divides to enhance understanding of human history [79,80]. Applying such frameworks to the interpretation of stable isotope data can improve insights into social negotiations that impact mobility and identity, as well as diet [81,82]. Multi-species anthropological perspectives in particular could be valuable interpretive lenses in stable isotope research for revealing the intricate relationships between humans, animals and their environments in such systems. When archaeofaunal remains are scarce, stable carbon and nitrogen isotope analysis of bone collagen provides a valuable proxy for reconstructing reindeer herding systems within specific environments and exploring interspecies interactions—particularly those that shape the community’s diet.

### (c) Rangifer tarandus

*Rangifer tarandus* (reindeer and caribou) are a medium-sized member of the Cervidae family and Arctic-adapted ungulates with a circumpolar distribution from the subarctic boreal forest to the high Arctic [83]. Their diet varies seasonally: in winter, they consume energy-rich, lichen-dominated forage, while in summer, they shift to a protein-rich diet consisting of fungi and large vascular plants such as herbs, shrubs and grasses [77].

During times of nutritional stress, which can be annual, reindeer also opportunistically consume bones, rodents, bird eggs, droppings and seaweed and kelp [84–87].

The migratory behaviour of *Rangifer* varies significantly depending on habitat, genetic lineage and environmental conditions. Some populations migrate extensively (50−1500 km), while others remain relatively sedentary, with movement patterns shaped by seasonal resource availability [88–90]. In spring, they frequently change pastures to follow the nutrient-rich new growth of plants, whereas in summer, movement is generally reduced [91,92]. The extreme climatic conditions of northern latitudes significantly influence their foraging strategies, as reindeer require high-quality forage to meet their physiological and metabolic demands [93,94]. This seasonal variation in diet and habitat drives the hyper-mobility of reindeer, with breeding females choosing higher-quality grazing areas to reduce nutritional stress [95].

In Arctic and subarctic regions, seasonal resource scarcity and nutritional stress shape the interactions between humans and animals [96–101], potentially influencing their cohabitation. Unlike other domesticated animals, reindeer follow similar seasonal cycles to their wild counterparts [102,103]. However, practices such as supplementary feeding or guiding reindeer to better pastures in North Asia may leave observable isotopic signals. These interventions benefit the reindeer and shape herders’ daily activities, reflecting multi-species relationships. Isotopic studies of *Rangifer* provide valuable insights into human–reindeer interactions, including seasonal diets, movement and herding strategies [77,104,105].

Different herding strategies may influence isotopic signatures, with free-ranging reindeer potentially exhibiting dietary variability owing to access to diverse plant communities, while more intensively managed herds could display narrower isotopic ranges, though individual feeding choices may also play a role. Isotope signatures could also help trace reindeer’s annual nutritional stress and community interventions to mitigate it, providing valuable insights into multi-species living. By comparing isotopic signals between herding systems, we can identify differences in seasonal movement, pasture management and anthropogenic influences, thereby providing deeper insights into how herders navigate environmental constraints and cultural practices in different ecological contexts.

### (d) Study area

**Figure 1.**
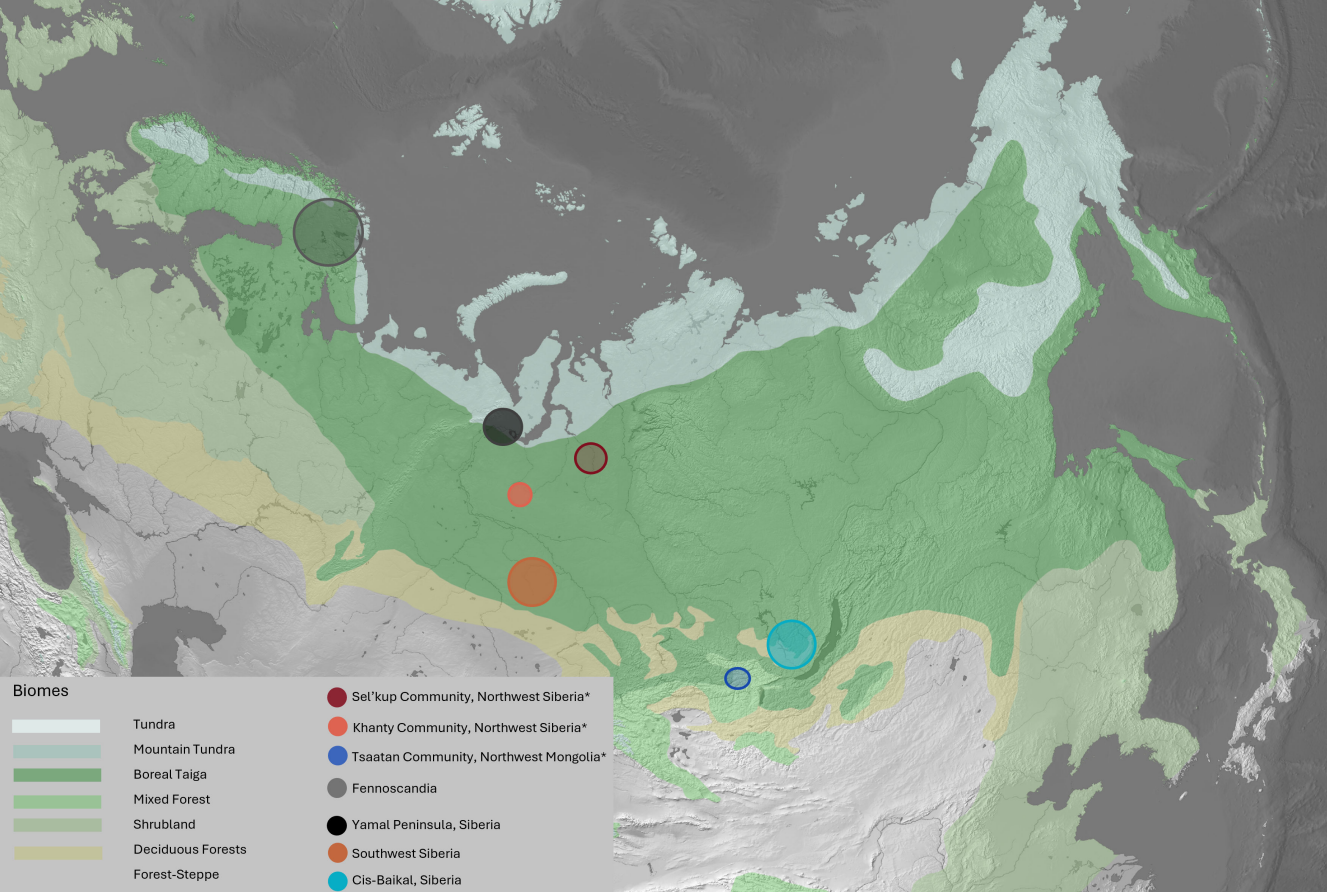
Location of study areas (Sel’kup community in west Siberia = red; Khanty = light orange; Tsaatan community in Khövsgöl, Mongolia = blue). Location of comparative studies from Fennoscandia, southwestern Siberia and Cis-Baikal Siberia, and the Yamal Peninsula (from studies [103,108–116]).

Where treeless tundra habitats dominate the Arctic, the Subarctic can be defined by the approximate latitudinal extents for boreal ecosystems [106]. These boreal regions, collectively the ‘taiga’ in Eurasia, are a complex mosaic of habitats, changeable landscapes and widely dispersed human settlements [107]. Taiga landscapes are rich in resources, characterized by mixed coniferous forest, lichen, wetland vegetation and a variety of berries and mushrooms. The wild fauna encompasses large Arctic ungulates (elk/moose, wild reindeer), carnivores including bears and wolves, smaller mammals, birds (such as forest fowl/grouse, ptarmigan, etc.) and freshwater fishes. The foci of this study are two small-scale herding groups (Sel’kup and Tsaatan) and, to a lesser extent, a third (Khanty). These communities, either currently or historically engaged in reindeer herding, inhabit regions within the past and present extent of the Eurasian boreal (taiga) forest (figure 1). In collaboration with these communities, we recorded herd management practices and sampled animal bones from these two regions. See the electronic supplementary material, appendix A, for specific environmental descriptions of the regions.

### (e) The Taz Sel’kup and Khanty of west Siberia

The northern Sel’kup are a subset of the Samoyedic language-speaking group whose name means ‘forest people’. They migrated north from the Tomsk region to the Taz River in the seventeenth and eighteenth centuries owing to settler-colonial pressures, evicting local Enets groups from the area [117] and adopting local subsistence and cultural traits [118]. After arriving in this region, the socio-cultural and economic practices of the Sel’kup changed [70], resulting in substantial overlap in cultural features between neighbouring groups, including the neighbouring Khanty. Historically, Khanty groups have engaged in small-scale reindeer herding and hunter–fisher migrations along the river valleys of the middle and lower Ob and Irtysh rivers [119]. This study includes one sample from Khanty contexts. The northern (Taz) Sel’kup’s primary adaptation to the area was the adoption of reindeer herding [117]; the incorporation of small-scale reindeer herding in which small herds of *ca* 10−70 head of deer are used almost exclusively for transport purposes, namely sled-pulling in bi-annual migrations to winter and summer campsites and during winter hunting expeditions, travelling along the frozen Taz River and into the tundra–taiga boundary area [70,118]. Their deer typically facilitate their fish–hunter–gatherer lifeways, slaughtering their domestic deer extremely rarely.

Taz Sel’kup use seasonal settlements and stations, with traditional dwelling structures including winter earth houses with ground-level floors (Sel’kup *poi-mot* = ‘wooden house’), conical tents (Russian *chum*) for both summer and winter, and more recently introduced wooden log houses (which are increasingly replacing traditional structures) [118]. Management of reindeer herds among Sel’kup communities is very free-range, with minimal controls placed on reindeer’s mobility, particularly in summer. A key management tactic is the use of smokehouses/outdoor ovens to encourage reindeer to stay near summer settlements during the most intense of the summer insect harassment, which is attuned to reindeer’s acute insect avoidance behaviours and encourages tameness and proximity to the summer settlement [117,119,120].

### (f) Tsaatan of the northwest Mongolian ‘taiga’

The forest zone in the far north of western Mongolia was historically the southernmost extension of the Eurasian taiga belt [121], is found around Lake Khövsgöl, the Darkhad Basin and the Sayan Mountains and extends into the Khentii mountains [122], forming a critical ecological zone. This taiga is divided into two, the Baruun (west) and Zuun (east) taiga, and is home to the reindeer herding Dukha ethnic group. The Dukha are ethnically Tuvan and share linguistic, cultural and historical ties with the broader Tuvan ethnic group in the Republic of Tuva, Russia, maintaining traditional practices that link them to their Siberian counterparts. Historically, they migrated through the Sayan Mountains until Soviet-era policies in the 1920s restricted their movement, and the closure of the Mongolia-USSR border in the 1940s further isolated them. By the 1980s, reindeer husbandry was nearly extinct, but efforts during Perestroika, combined with the importation of Siberian reindeer and veterinary reforms, helped revive the practice [123–126]. The Tsaatan community today constitutes around approximately 30 households/families, with approximately 500 people continuing the Tuvan traditional lifeways as pastoral people, living in conical *urts* [124,127]. Their reindeer husbandry today is part of an integrated economic mosaic that features aspects of both taiga- and steppe-based pastoralism, as well as small-scale hunting, fishing, gathering, agriculture, inter- and intra-regional trade and remunerated labour [125,126]. In the last century, Tsaatan lifeways have been strictly limited by the creation of national borders and the creation of the Ulaan Taiga Special Protected Area (UTSPA) [128].

## 2. Methods

### (a) Ethnographic fieldwork

Fieldwork was conducted over multiple seasons from 2016 to 2023 (see the electronic supplementary material, appendices A and C), with a focus on documenting herding practices and their archaeological traceability. The team visited Taz Sel’kup summer and abandoned winter settlements, various Kazym Khanty communities in west Siberia, Russia and Tsaatan seasonal camps in the Baruun taiga, Mongolia. In addition to ethnographic methods like participant observation and informal interviews, the research included the collection of modern and recently excavated bone samples from summer settlements and camps. These samples, primarily from herded reindeer, were analysed to assess dietary patterns and husbandry practices. Local participants, either current or former herders, played a key role in guiding the research, which also involved archaeological surveys, photography and excavations, all conducted in collaboration with community partners (see the electronic supplementary material, appendices A and C, for full breakdown for field methods and sample origins).

## 3. Materials

To situate reindeer stable isotopic values not only within the two specific taiga environments, but also the larger food web, a range of other animal species (mammals, birds, fishes) were also sampled to better contextualize results and account for possible anthropogenic influence. We analysed samples from 34 animals: *Alces alces* (*n* = 5), *Falcipennis falcipennis* (*n* = 1), *Lepus timidus* (*n* = 4), *Esocidae/Percidae* sp. (*n* = 5) and *R. tarandus* (*n* = 19). We collected surface-scattered *Rangifer* bones from two Sel’kup summer settlements (*n* = 5), one Khanty summer settlement (*n* = 1) and four Tsaatan campsites (*n* = 10) (see the electronic supplementary material, appendix C, tables S1–S3).

Surface scatter reindeer bones were herded reindeer, according to community members. Two individuals were recently deceased herd members with known sex, age and health data, one of which (KIKK 122) was buried after death to avoid attracting predators to the area (see the electronic supplementary material, appendix B, figure S7).

Over the course of fieldwork in 2021, in addition to ethnographic activities, we excavated a semi-subterranean *poi-mot* on the Taz River which dated to the 1920s based on Sel’kup families and elders whose summer settlement was located next to the *poi-mot* and associated finds. Animal bones were present in two areas of the excavation, two pits on either side of the house platform, and all the seven samples came from pit 1 (see the electronic supplementary material, appendix B, figure S6). Only the *L. timidus* and three *R. tarandus* samples were from the 1920s *poi-mot*. With the exception of the 1920s samples, where the lifestyle of the reindeer and herders cannot be confirmed, all reindeer samples from west Siberia and Mongolia were from herded reindeer. All samples were collected over a series of ethnographic fieldwork seasons and contextualized through the documentation of herding practices (see the electronic supplementary material, appendix A).

### (a) Sample selection

During the lifetime of reindeer, their bones are remodelled, like any animal [129], but nonetheless represent overarching trends in diet, whereas other tissues like antler isotopic values cannot represent more than a year of a reindeer’s life owing to annual shedding [75]. For these reasons, all samples came from adult animals to reduce potential variations driven by younger animals’ bone turnover or nursing-related dietary signatures and to ensure consistency in time averaging. Where the age and sex of individuals were not known (via herder), adulthood was determined by confirming bones were fully fused and through tooth wear (particularly where the M3 was fully erupted and in wear; see the electronic supplementary material, appendix C, table S2). We excluded ribs and antlers from the range of skeletal elements, opting instead for mandibular bone and post-cranial limb bones which have lower turnover rates [130,131]. See the electronic supplementary material, appendix C, tables S2 and S3, for full list of sample, ages and values.

### (b) Sample preparation and analysis

As all samples (surface collected, buried or ethnoarchaeological) date from the 1920s to 2022 and are intended for comparative analysis between modern and archaeological samples, the Suess effect was considered. The Suess effect suggests that an overall dilution of atmospheric ^13^CO_2_ is the result of large amounts of fossil-fuel-derived CO_2_ depleted in ^13^CO_2_ admixing into the atmosphere [132]. Following [133], carbon results from samples from the 1920s *poi-mot* were adjusted, adding +0.5‰ and +2.0‰ to results from surface remains collected between 2020 and 2022, as animals with known year at death were within this data range, and bones collected from the surface are expected to be from within the last 5 years (see the electronic supplementary material, appendix B, table S3).

Samples were prepared and analysed at the Curt-Engelhorn-Zentrum Archäometrie gGmbH (CEZA). Demineralization was over 2−4 weeks in 10 ml of 0.5 M hydrochloric acid (HCl), first at 4°C and later at room temperature, followed by the removal of the surface and cancellous bone material with a dental drill. The acid solution was changed every 7 days until complete demineralization then rinsed to pH neutrality. As the samples were surface finds or buried bones humic acid removal was carried out in 10 ml of 0.1 M NaOH overnight at approximately 4°C, with the removal of NaOH through rinsing until pH neutrality. Collagen samples were then gelatinized in 4 ml of 0.001 M HCl (pH 3; 70°C, max. 48 h). Removal of insoluble particles was completed using Ezee filters (Elkay; pore size approx. 60−90 µm) and lyophilization was completed over 48 h. Collagen samples were weighed into tin capsules (between 680 and 730 µg), and mass spectrometry was performed on triplicate samples. A vario ISOTOPE select elemental analyser (Elementar GmbH, Langenselbold) in carbon, nitrogen, and sulfur (CNS) mode at CEZA was used for combustion and determination of the carbon and nitrogen contents. Calibration of the element contents used internal software based on the calibration standards for elemental levels: sulfanilamide, United States Geological Survey (USGS) 43, USGS 40 and USGS 41a. An Isoprime visION isotope ratio mass spectrometer (Elementar GmbH, Langenselbold) determined the isotope ratios *δ*^15^N and *δ*^13^C. An internal software calibrated the results using reference standards IAEA-CH-6, IAEA-CH-7, IAEA-N-1, IAEA-N-2, USGS 43, USGS 40 and USGS 41. Results use the delta notation in parts per thousand (‰) relative to the international standards Vienna PeeDee Belemnite (VPDB) for carbon and atmospheric nitrogen (AIR) for nitrogen.

The atomic C : N ratio is commonly used as a criterion for the degree of preservation of collagen purified for radiocarbon dating and stable isotope analysis: values between 2.9 and 3.6 are accepted as indicators of good collagen preservation [134,135], values outside this range were excluded from analysis (electronic supplementary material, table S2, appendix B).

We did not perform a lipid removal step because the skeletal remains were surface finds that had already undergone significant weathering leading to natural lipid degradation, and additional processing could have further compromised collagen integrity (see the electronic supplementary material, appendix A). Our elemental and isotopic data show no signs of lipid-related bias, and since comparative reindeer datasets (see [103]) also did not include lipid extraction, our results remain consistent and directly comparable to other published data.

### (c) Statistics

The data results were checked for normality with the Shapiro–Wilk test using PAST software [136]. Student’s *t*-tests were used when the means of two groups were tested for variance and normality was found. When more than two groups were compared, a one-way analysis of variance was followed by post hoc comparisons using Tukey range tests on all possible pairwise combinations. Differences were considered significant for *p*‐values <0.05. Non-parametric tests compared our results for variance against published datasets, as normality was not found in all comparative regional datasets. When comparing our results against these external datasets Kruskal–Wallis and Dunn’s *post hoc* pairwise combination comparisons were used. See the electronic supplementary material, appendix B, table S4, for all statistical results.

## 4. Results

### (a) Ethnography

#### (i) Taz Sel’kup and Khanty

The Taz Sel’kup families we visited and collected samples from move twice a year between their summer and winter settlements, which are 8−20 km apart in the taiga, staying along the Taz River and its tributaries. That said, with the use of boats and extensive riverine systems, they are highly mobile and equally allow their reindeer free range. They indicated that past generations migrated biweekly to monthly before Sovietization and the introduction of log houses. In addition to their summer and winter housing, other buildings on the summer settlements include stilted storage houses. Of particular note for this study are (i) reindeer smoke infrastructure and (ii) wooden storage containers, particularly for storing fish to feed the multi-species community in winter, including the reindeer (electronic supplementary material, appendix B, figure S2).

**Figure 2.**
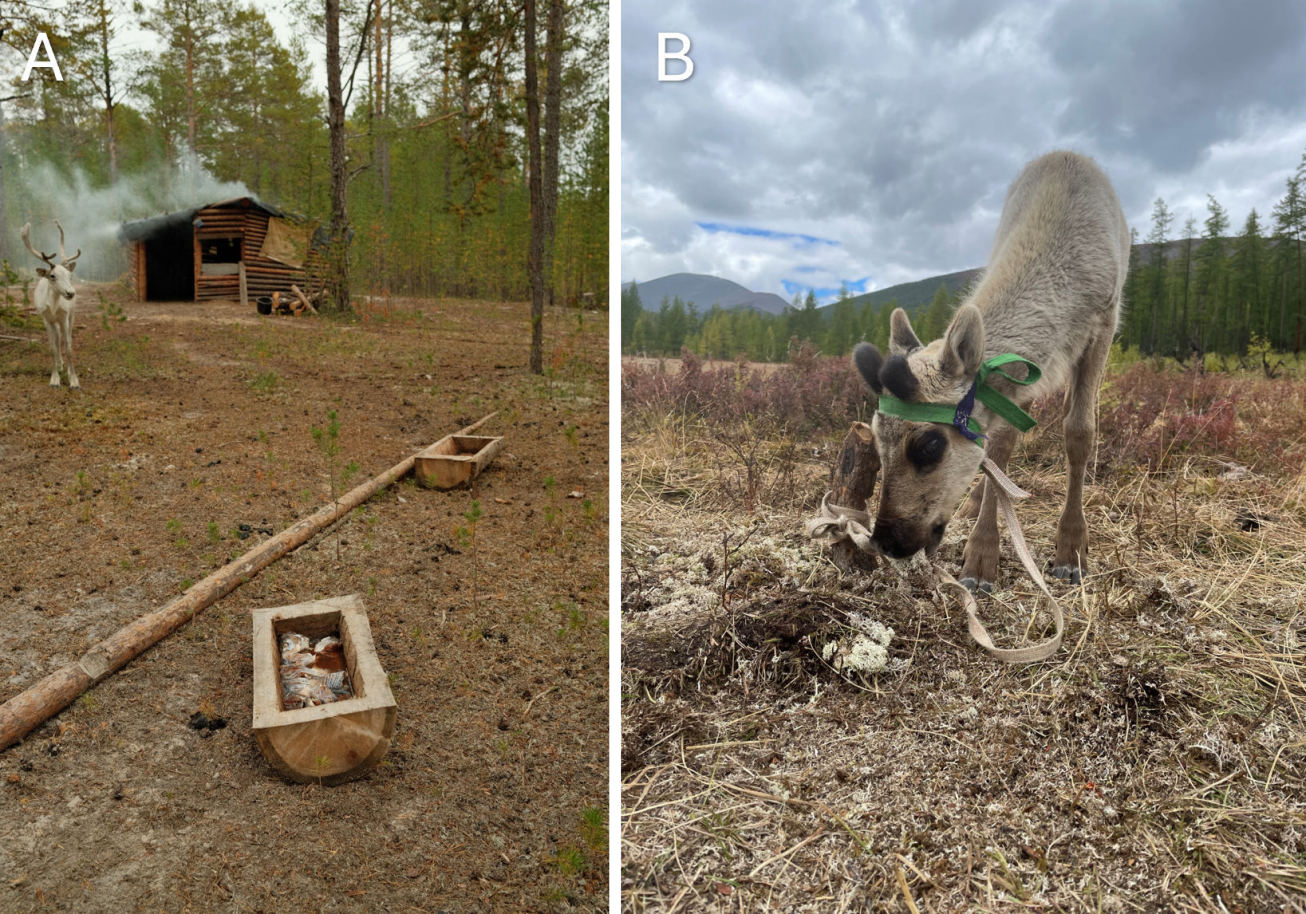
Examples of reindeer feeding across the study areas. (A) Fish feeding in troughs near reindeer house architectures among Sel’kup communities, and (B) lichen gathering and feeding to deer among Tsaatan. Photos by M. Windle.

These constructions are specifically built and used for the small-scale reindeer husbandry in connection with the insects, namely ‘smoke houses’ as well as one or two smoke ovens inside. Alongside the smoke infrastructure is the placement of fish troughs by the houses and fires, which reindeer can consume throughout the day (figure 2).

Supplementary feeding was indicated as common among Sel’kup herders, primarily through fish feeding, both with troughs near smoke houses and hand feeding. The practice of fish feeding occurs year-round, according to the families we visited, to the extent that particular wooden storage facilities are built for storing fish for the deer. In our experience, fish feeding is done near to the smoke infrastructure to encourage the use of the smoke infrastructure, increase tameness around people, and provide extra nutrients to the deer. The fish feeding varied depending on the family, with two families tending to put fish in troughs on a weekly to monthly basis in summer and daily in the winter. By contrast, another family fed fish to their reindeer daily, with constant maintenance of the troughs, all year round. Procuring the fish is done in tandem with the daily fishing of Sel’kup family members to meet their own dietary needs, and typically the species of fish was the same as what was caught for the families, with the most common being pike (*Esox* sp.)*.* Certain species are intentionally excluded and not fed to reindeer, namely spiny-finned fish such as perch (*Perca* sp.) *as t*heir spikes can upset the reindeer’s stomachs.

The inclusion of the Khanty sample is used here because while the specimen was from a herded reindeer (according to the Khanty family), the community did not feed fish to their deer. This sample therefore represents the west Siberian taiga biome but without the influence of fish feedings, compared to samples from Sel’kup settlements.

#### (ii) Tsaatan (Dukha)

Tsaatan families we visited and collected samples from rely almost exclusively on their reindeer to facilitate their nomadic lifestyles, using them both as pack and riding animals for seasonal migrations and as everyday transport when herding deer or visiting neighbours. Additionally, reindeer milk serves as an important food resource. Tsaatan herded their reindeer daily to pasture, staking them near the *urts* during the night when they were not grazing. Reindeer management also involves practices such as hobbling or tying pairs of deer together at their antlers to limit mobility and simplify herding (electronic supplementary material, appendix B, figure S3). Seasonal camps are typically located in lower-elevation valleys or relatively flat, high-elevation terraces. Interviews suggest that open-air smoke infrastructures were used, similar to those of the Sel’kup, but this practice has fallen out of favour owing to declining insect populations. Instead, mobility plays a central role in their management, with reindeer husbandry practices built on the relationships between insects and reindeer.

Tsaatan families’ mobility is highly variable, with camps being moved biweekly, monthly or seasonally. These moves typically occur between mountain valleys and the high-alpine tundra, more commonly in spring, summer and autumn. In winter, male family members cooperate in herding their deer. To supplement their reindeer’s diet, they gather lichen throughout the year, with targeted lichen gathering occurring in winter. During this time, they do not migrate but instead move their herds between lichen fields and the winter settlement, where lichen is routinely gathered. When asked about intentionally feeding their reindeer, Tsaatan community members mentioned that they occasionally gather lichen from higher elevations in summer for young or injured deer (figure 2). By contrast, herders routinely collect and pile lichen for daily consumption by the reindeer in the winter. One family also indicated that urine (collected in pits or containers) is used year-round to attract deer near the *urts*, as it provides essential salt and minerals.

### (b) Isotopic results

Of the 34 samples analysed, 29 fell within the accepted range of collagen of 2.9–3.6, whereas all fish samples had a range of C : N ratios from 3.8 to as high as 5.2, indicative of diagenic changes and rendering them unreliable (electronic supplementary material, appendix B, table S2). No samples warranted exclusion owing to unreliable readings from the elemental analyser.

**Figure 3.**
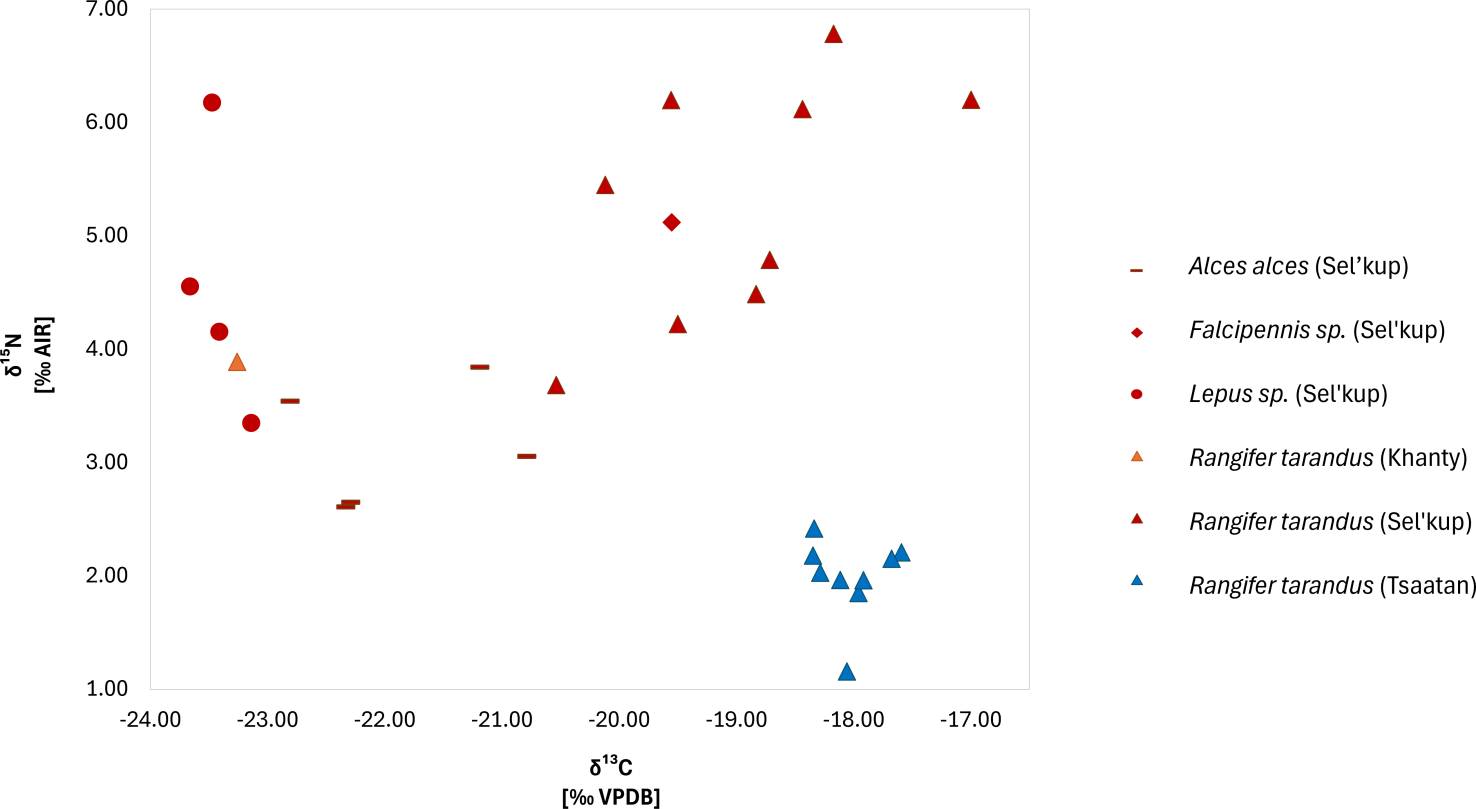
Carbon and nitrogen isotopic values from Sel’kup and Khanty communities, West Siberia and Tsaatan communities, northwest Mongolia.

The corrected for Suess effect *δ*^13^C values of samples from west Siberia ranged from −23.8 to −16.9‰ (mean −20.8 ± 2.4 ‰; figure 3). The *δ*^13^C values of free-ranging domesticated reindeer ranged from −23.3 to −16.9‰ (mean −19.3 ± 2.3‰) and the single sample from the Khanty herd had the lowest carbon value. The range for other species from the Sel’kup area was as follows: −23.8 to −21.2‰ (mean −22.53 ± 1.1‰) for *A. alces;* −23.5 to −22.2‰ (mean −23.05 ± 0.6‰) for *Lepus* sp. *s*amples; and the single *F. falcipennis* sample had a value of −19.6‰. In contrast to the west Siberian samples, Tsaatan reindeer had a far narrower range of carbon values from −18.4 to −17.6‰ (mean −18.1 ± 0.3‰) with, notably, a higher median than the west Siberian samples.

The *δ*^15^N values of samples from West Siberia ranged from 2.6 to 6.8‰ (mean 4.5 ± 1.3‰; figure 3). The reindeer ranged from 3.7 to 6.8‰ (mean 5.2 ± 1.1‰) and the single sample from the Khanty herd was in the lower range with a value of 3.9‰. The range for other species from the Sel’kup area was as follows: 2.6 to 3.8‰ (mean 3.2 ± 0.6‰) for *A. alces;* 3.3 to 6.2‰ (mean 4.6 ± 1.2‰) for *Lepus* sp. samples; and the single *F. falcipennis* sample had a value of 5.1‰. As with the carbon values, Tsaatan reindeer had a far narrower range of nitrogen values from 1.2 to 2.4‰ (mean 2.0 ± 0.4‰).

Student’s *t*-tests yielded a *p*-value of 2.44 × 10^−7^ between nitrogen values and a *p*-value of 0.027309 for carbon (electronic supplementary material, appendix B, table S4) of the two regions, demonstrating a significant difference between the west Siberian and Mongolian reindeer herds. Two potential clusters within the Sel’kup reindeer samples were identified (one cluster with lower *δ*^15^N values, *n* = 4, and higher *δ*^15^N values, *n* = 5, respectively), which we tested for significant difference using a Student’s *t*‐test. The intragroup clusters yielded a *p*-value of 0.0002 for stable isotopic nitrogen, demonstrating a statistically significant difference in the latter between the two Sel’kup reindeer clusters.

## 5. Discussion

**Figure 4.**
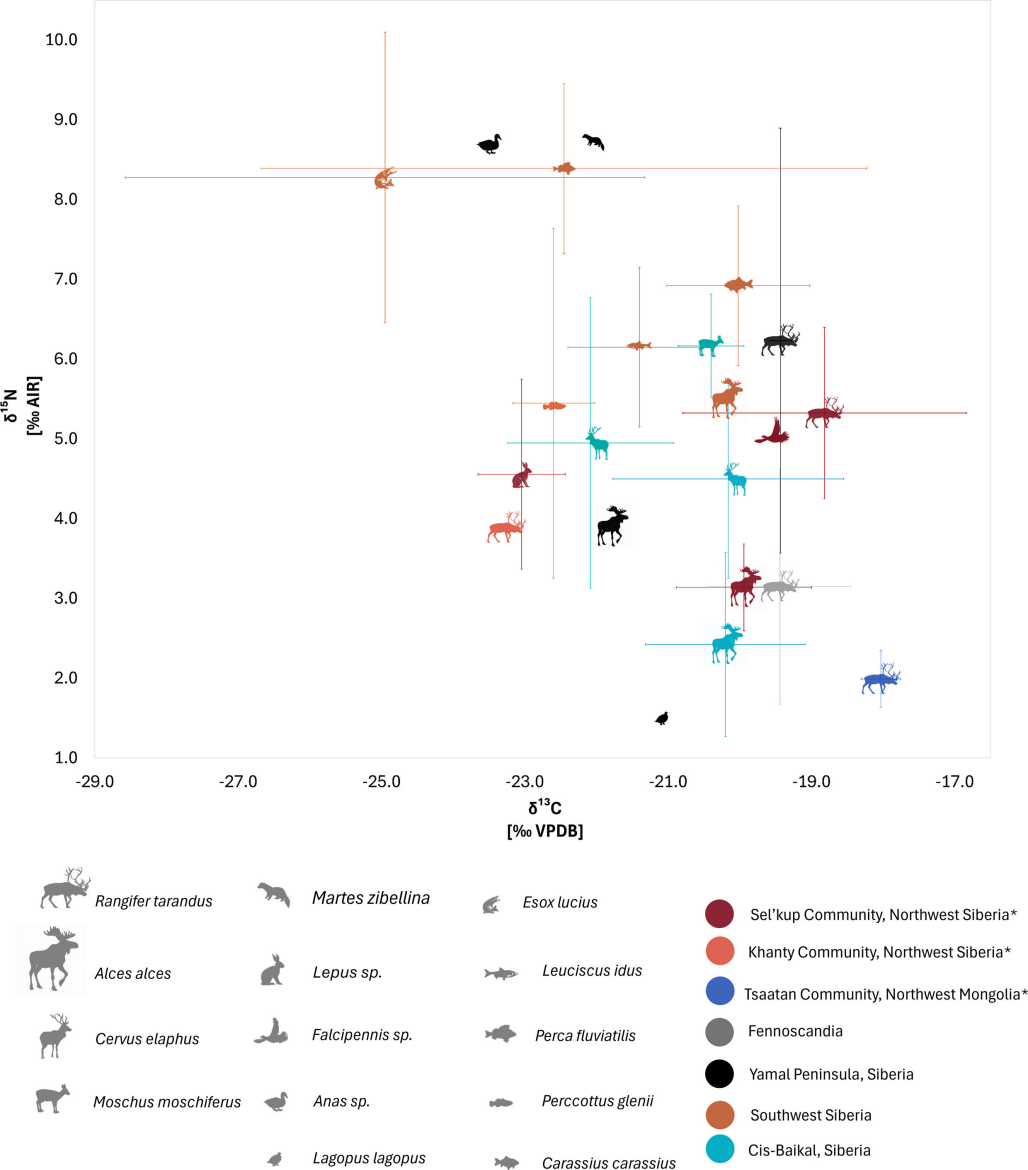
Summary graph showing the mean and standard deviation of *δ*^13^C and *δ*^15^N values from west Siberia* and Mongolia* and from Fennoscandia, southwest Siberia, Cis-Baikal Siberia, and the Yamal Peninsula, Siberia datasets (using data from studies [103,108–116]). *Original data.

To help contextualize our results, we compiled published *δ*^13^C and *δ*^15^N values from regions where reindeer herding occurs and similar regional taxa in the study areas (figure 4; [103,108–116]). All modern comparative values were corrected for the Suess effect in their respective studies. However, we excluded non-adult, rib and antler samples to ensure better comparability with our dataset. Comparative reindeer samples are a combination of archaeological and modern contexts, the latter being from free-range, zoo and herded contexts [103,109,112,114–116]. In Fennoscandia, existing archaeological reindeer samples (*ca* 1200−1700 CE; [103,114–116]) had a *δ*^13^C range of −22.0 to −17.7 (mean −19.4 ± 1.6‰) and modern reindeer −21.0 to −18.3‰ (mean −19.7 ± 0.8‰; [103,112]). *δ*^15^N values were 1.1 to 8.5‰ (mean 3.4 ± 1.6‰). *δ*^15^N values were 1.1 to 8.5‰ (mean 3.4 ± 1.6‰).

Archaeological isotopic data from Siberian reindeer are limited, with available data from the Yamal Peninsula’s Uist Polui site [109]. Here, *δ*^13^C values ranged narrowly between −19.6 and −19.3‰ (mean −19.4 ± 0.2‰), and *δ*^15^N values spanned between 3.3 and 8.5‰ (mean 6.2 ± 2.7‰). We tested nitrogen variance between our west Siberian results and both the Fennoscandian and Yamal Peninsula data, finding significant differences (*p* = 1.43 × 10^−5^), specifically between our west Siberian, Mongolian and Fennoscandian reindeer datasets, as shown by Dunn’s *post hoc* (electronic supplementary material, appendix B, table S4). However, no significant difference was found between west Siberian and Yamal samples (*p* = 0.9), though the Yamal data included only a few samples (electronic supplementary material, appendix B, table S4).

### (a) Carbon

The *δ*^13^C range among Siberian samples probably reflects the diverse species and biomes of their respective origins across the Cis-Baikal, Arctic tundra, and taiga regions of North Asia (figures 1 and 3). Notably, Mongolian Tsaatan reindeer had slightly higher *δ*^13^C values (mean −18.1 ± 0.3‰) than their Siberian counterparts (mean −19.3 ± 2.3‰). This is probably, in part, owing to baseline isotopic differences between the C3-dominant ecosystems, shaped by local temperature and water availability. Regional plant isotope studies support these C3-specific variations, with taiga environments in Siberia (Sel’kup) characterized by marshy landscapes, while Mongolian (Tsaatan) samples originate from drier, mountainous regions.

The fish feeding we documented among Sel’kup herders could also influence *δ*^13^C values in reindeer. Freshwater fishes exhibit highly variable *δ*^13^C signatures depending on the aquatic ecosystem, ranging from values like terrestrial C3 plants to significantly enriched levels depending on a fish species’ diet [137–140]. If fish feeding was substantial enough to impact *δ*^15^N values, it is likely that it also influenced *δ*^13^C values in the Sel’kup samples. That said, the precise influence on carbon cannot be easily disentangled from other influences using bulk collagen. Future studies incorporating isotopic baselines for local fish species could clarify this potential dietary contribution. Additionally, lichen consumption could significantly influence the reindeer *δ*^13^C values. Lichen typically exhibits enriched *δ*^13^C signatures compared to vascular plants, meaning that reindeer consuming high proportions of lichen could show slightly elevated *δ*^13^C values relative to those primarily feeding on C3 vegetation [112,113]. Considering the practice of intentional gathering of lichen throughout the year to feed to their deer in Mongolia, though especially in winter, this could explain the slightly elevated *δ*^13^C values among the Mongolian reindeer.

Herding strategies probably influenced the isotopic variation as well. In Siberia, Sel’kup reindeer herding involves minimal management, with herders rarely directing animals to specific pastures. Instead, they rely on smokehouses to guide the reindeer back to summer settlements (electronic supplementary material, appendix B, figure S1). This free-range approach allows animals to access diverse landscapes and plant varieties within the taiga. By contrast, Tsaatan herders in Mongolia operate within the UTSPA, which, while expansive, may offer different ecological conditions compared to broader free-ranging areas. Despite the spatial extent of the UTSPA, Tsaatan herders restrict reindeer mobility by hobbling pairs of reindeer, tying them to limit their range and guiding them to specific pastures near their summer campsites. This directed grazing strategy could reduce exposure to diverse vegetation types compared to Siberian herds. Additionally, while the UTSPA encompasses extensive terrain, and seasonal pasture rotation distances can be considerable, the pastures within park boundaries may still show limited vegetation diversity [141–143]. Expanding research on pasture degradation, particularly within restricted areas like the UTSPA, would enhance our understanding of stable isotopic carbon patterns and their link to herding practices.

### (b) Nitrogen

The dramatic environmental changes (such as shifts in temperature and altitude) that reindeer experience owing to their grazing and migratory behaviours can influence their isotopic signatures. A primary example is the inverse relationship between altitude and isotopic values, where decreased lichen consumption lowers *δ*^13^C values, while higher altitudes correspond with decreased *δ*^15^N and increased *δ*^13^C values [144]. ^15^N values may also be affected by differences between Arctic and subarctic lichens and vascular plants [145,146]. While it is currently unclear how intra- and inter-annual variation in body *δ*^15^N values (measured from blood, muscle and urine) translate into bone *δ*^15^N values [147,148], our results reflect the different herds’ environmental and dietary influences over several years.

Nutritional stress and starvation, which lead to the re-use of muscle protein, result in higher *δ*^15^N [149]. As mentioned, this is common for reindeer in winter owing to limited access to high-quality graze [150,151]. However, it is unlikely that this effect alone accounts for the elevations observed in the Sel’kup samples, as seasonal nutritional stress would homogenize during bone collagen remodelling, resulting in lower overall enrichment signatures [104]. Year-round fish feeding, as documented in the Sel’kup case, may contribute to *δ*^15^N enrichment. When consulted alongside existing fish baselines (figure 4), fishes in Siberia exhibit a broad *δ*^15^N range, overlapping with both omnivorous mammals and the Sel’kup reindeer samples (figure 4).

Our fieldwork showed that Sel’kup families do not engage in plant foddering but instead practise direct feeding. Although fish feeding has frequently been recorded as a winter practice [152], we found that it can occur year-round. Herders are acutely aware of their reindeer’s nutritional needs and the frequent winter stress caused by extreme conditions. To mitigate this stress, they provide fish as a food source, either by placing troughs near smokehouses or by handfeeding (figure 2; also the electronic supplementary material, appendix C, figure S2). Beyond its nutritional role, ethnographic accounts suggest that fish feeding may also function as a herding technique to attract and tame reindeer, similar to the use of smoke or salt in reindeer management [2,153]. These overlapping motivations highlight the complex interactions that shape Sel’kup herding practices.

Comparing these findings to other managed reindeer populations provides further context for understanding isotopic enrichment. Elevated nitrogen values in modern Fennoscandian reindeer have been linked to industrial hay foddered with organic fertilizer [102]. In archaeological reindeer (thirteenth century CE), similar elevations were suggested to be the result of winter foddering of the reindeer with grasses, sedges and dried tree branches [103]. The mean *δ*^15^N values from Fennoscandian studies (figure 4), interpreted as elevated, are statistically significantly lower than our Sel’kup samples as well as the archaeological reindeer bones samples from Uist Polui in the Yamal Peninsula, Siberia (figure 4). Compared to the Fennoscandian *δ*^15^N values, the reindeer also sampled from Uist Polui are significantly enriched.

Notably, Losey and colleagues suggest that elevated *δ*^15^N values in dogs may reflect human provisioning of fish [108]. Similarly, historical and modern fish storage practices among reindeer herders, including drying and smoking or preparation of fish soup [153], ensure a steady supply for both human and animal consumption. The persistence of these practices, as reflected in our ethnographic findings, suggests that fish feeding may have long-shaped isotopic signatures and remains integral to reindeer herding in North Asia.

The routine consumption of faunal proteins through the practice of fish feeding could be an important factor contributing to elevated values we see in Siberia, particularly when contextualized alongside nitrogen values of modern and archaeological fish (figure 4) from nearby regions. We suggest here that the elevated values of our Sel’kup reindeer samples are a result of this routine supplemental feeding of fish proteins by herders to reindeer and subsequently represent an indicator of the multi-species living in the region.

Finally, the *δ*^13^C and *δ*^15^N differences between Tsaatan and Sel’kup reindeer reflect both diet and herding strategies. Tsaatan reindeer, managed within defined herding areas, show a narrower isotopic range, while free-ranging Sel’kup reindeer, with access to diverse forage and supplemental fish feeding, exhibit greater variation. Hobbling in Tsaatan herding may influence isotopic diversity, while Sel’kup mobility increases the exposure to varied vegetation and protein sources, potentially elevating *δ*^15^N values. These findings highlight how herding practices interact with dietary intake and isotope compositions.

### (c) Isotopes as an indicator of multi-species lifeways

**Figure 5.**
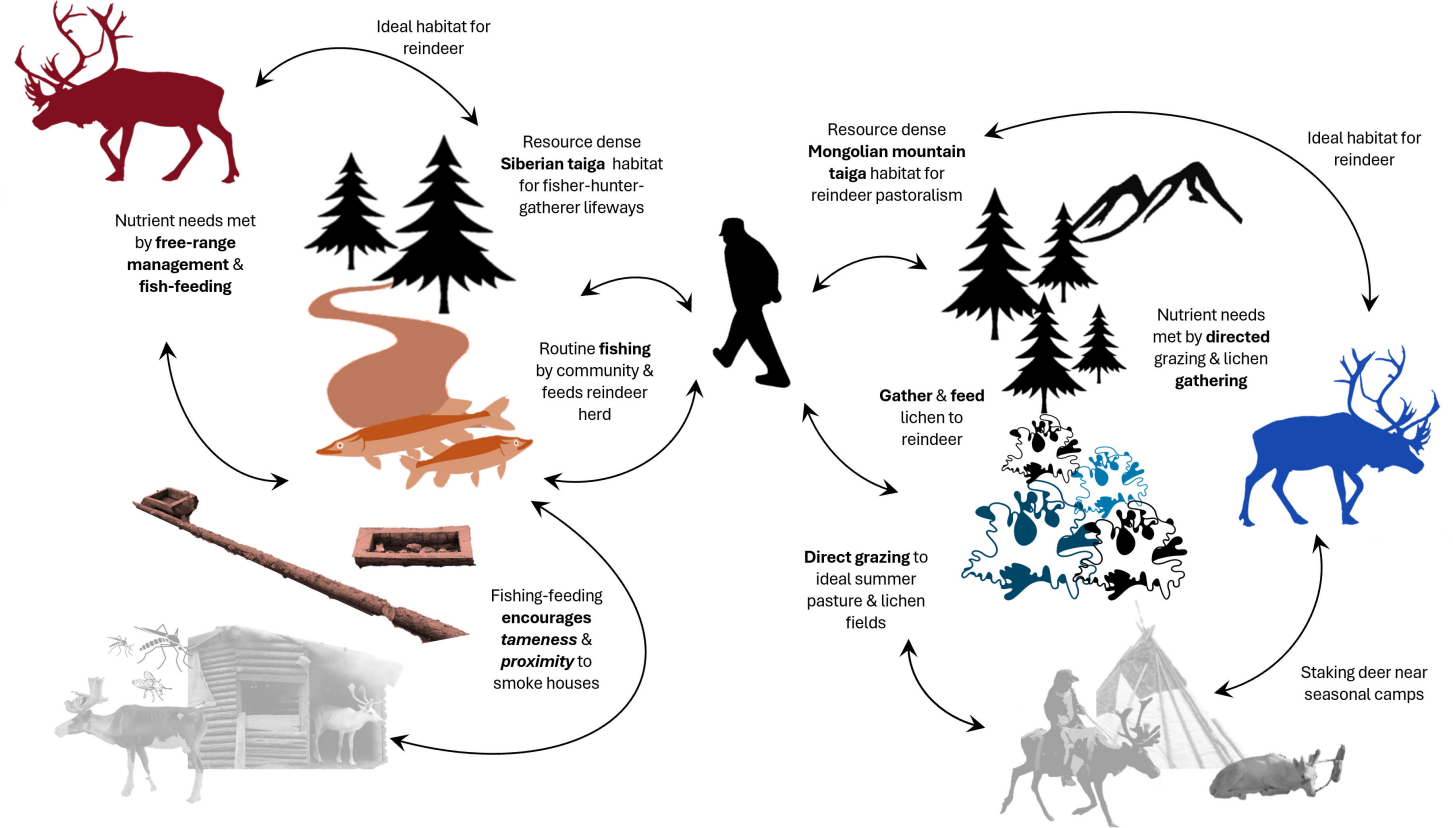
Tanglegram of described reindeer herding multi-species practices (red = est Siberia, blue = Mongolia).

Our results also show how isotopes can be representative of a nexus of multi-species actors and practices that can create a community (figure 5). In this case, a reindeer–fish–human interface co-creates the isotope record we encounter. Coupled with the mechanism that drives this, nutritional stress could have significant implications for understanding how reindeer domestication came to be. Notably, the influence of herding strategies, such as the directed grazing and mobility of Tsaatan reindeer versus the free-ranging Sel’kup herds, further shapes these isotopic signatures. The relationship between directed grazing, dietary diversity and supplemental feeding practices underscores the complex interplay between ecological adaptation and human management. Our understanding of animal responses to supplemental feeding is rapidly expanding, with studies demonstrating its long-term impacts on bird population dynamics and distribution [154], population densities, artificial selection of behavioural traits and herd health of ungulates [155,156]. Exposure to environmental and feeding conditions outside their normal habitat has led to extensive artificial selection across various orders, affecting Mongolian sheep [157]. Compensatory feeding like supplementing fish into the diet can have important implications. This is because through the combination of herder ecological knowledge, environment, and non-human species ecology, this specific niche-constructing practice arises and creates a feedback loop that encourages cooperation between herders and reindeer. This could then impact the selection pressures and subsequent domestication pathways for reindeer. Future directions should include larger studies of herder reindeer diets, look to other proteinaceous tissues to investigate the temporality of dietary shifts and incorporate other reindeer populations and herding communities across North Asia to expand on these results.

## 6. Conclusion

Stable isotope analysis is a powerful method for investigating human–animal interactions, particularly in regions with limited archaeological evidence. By examining stable carbon and nitrogen isotopes from reindeer bones, we can reconstruct dietary habits, mobility patterns and herding strategies, revealing the intricate multi-species relationships that shape reindeer herding systems. Our findings indicate that supplementary feeding practices, such as providing fish, leave distinct isotopic signatures that reflect human-mediated interactions with reindeer and their environments. Moreover, differences in herding strategies—such as the directed herding of Tsaatan pastoralists versus the free-ranging approach of Sel’kup herders—further influence isotopic variability by affecting dietary intake. These patterns underscore the role of multi-species interactions, where nutritional stress and compensatory feeding create feedback loops that may influence selection pressures and domestication pathways. This highlights the need for further research on reindeer diets across diverse populations and protein sources to better understand dietary shifts over time. By integrating multi-species perspectives, stable isotope analysis provides a critical lens for interpreting how human–animal relationships actively shape archaeological records, offering valuable insights into reindeer domestication.

## Data Availability

Data can be accessed in the electronic supplementary material [158].
